# Case Report: Pemetrexed-induced pseudocellulitis: a rare adverse effect to be recognized for better management

**DOI:** 10.3389/fonc.2025.1619250

**Published:** 2025-10-08

**Authors:** Emma Proux, Jeanne Chen, Caroline Bonhomme, Pierre Pottier, Marie Piroth

**Affiliations:** ^1^ Dermatology Department, University Hospital of Nantes, Nantes, France; ^2^ Department of Medical Oncology, University Hospital of Nantes, Nantes, France; ^3^ Department of Internal Medicine, University Hospital of Nantes, Nantes, France; ^4^ Department of Rhumatology, University Hospital of Nantes, Nantes, France

**Keywords:** pemetrexed, drug eruptions, pseudocellulitis, antifolate, cellulitis, pemetrexed-induced pseudocellulitis, drug-induced

## Abstract

Pseudocellulitis, a non-infectious inflammatory reaction mimicking infectious cellulitis, is a rare and often underrecognized adverse reaction to pemetrexed, frequently diagnosed late and leading to inappropriate treatments, particularly unnecessary antibiotic use. Through the presentation of a new case and a literature review conducted using Scopus and PubMed, we aim to clarify its clinical presentation and management. We report a new case of pemetrexed-induced pseudocellulitis (PIP), initially misdiagnosed and treated unnecessarily with multiple antibiotics. Following the correct diagnosis, the patient was successfully treated with both oral and topical corticosteroids. Pemetrexed had to be discontinued. PIP clinically mimics cellulitis, presenting unilaterally or bilaterally, sometimes accompanied by fever and inflammatory syndrome. Its onset is variable, occurring either after the initial pemetrexed administration or following subsequent cycles, with no clear dose dependency. Skin biopsy is not essential for diagnosis. Management typically involves local and/or systemic corticosteroids. Discontinuation of pemetrexed should be evaluated on a case-by-case basis and is not always necessary. Improved recognition of this condition is essential to avoid unnecessary interventions, enhance patient care, and prevent long-term complications due to prolonged inflammation.

## Introduction

Pemetrexed is an antifolate agent used in the treatment of non-small cell lung cancer (NSCLC) and malignant pleural mesothelioma (1, 2). Pseudocellulitis refers to a non-infectious inflammatory reaction that mimics infectious cellulitis, leading to diagnostic delays and multiple unnecessary courses of antibiotics. Cutaneous adverse effects of pemetrexed are common, ranging from mild erythema to severe toxic epidermal necrolysis (3), but pseudocellulitis was not reported in clinical trials (4). Through the presentation of a new case, diagnosed lately and treated with multiple lines of unnecessary antibiotics, and a review of the literature conducted on Scopus and PubMed, we aim to clarify its clinical presentation and management.

## Case report

**Figure 1:**
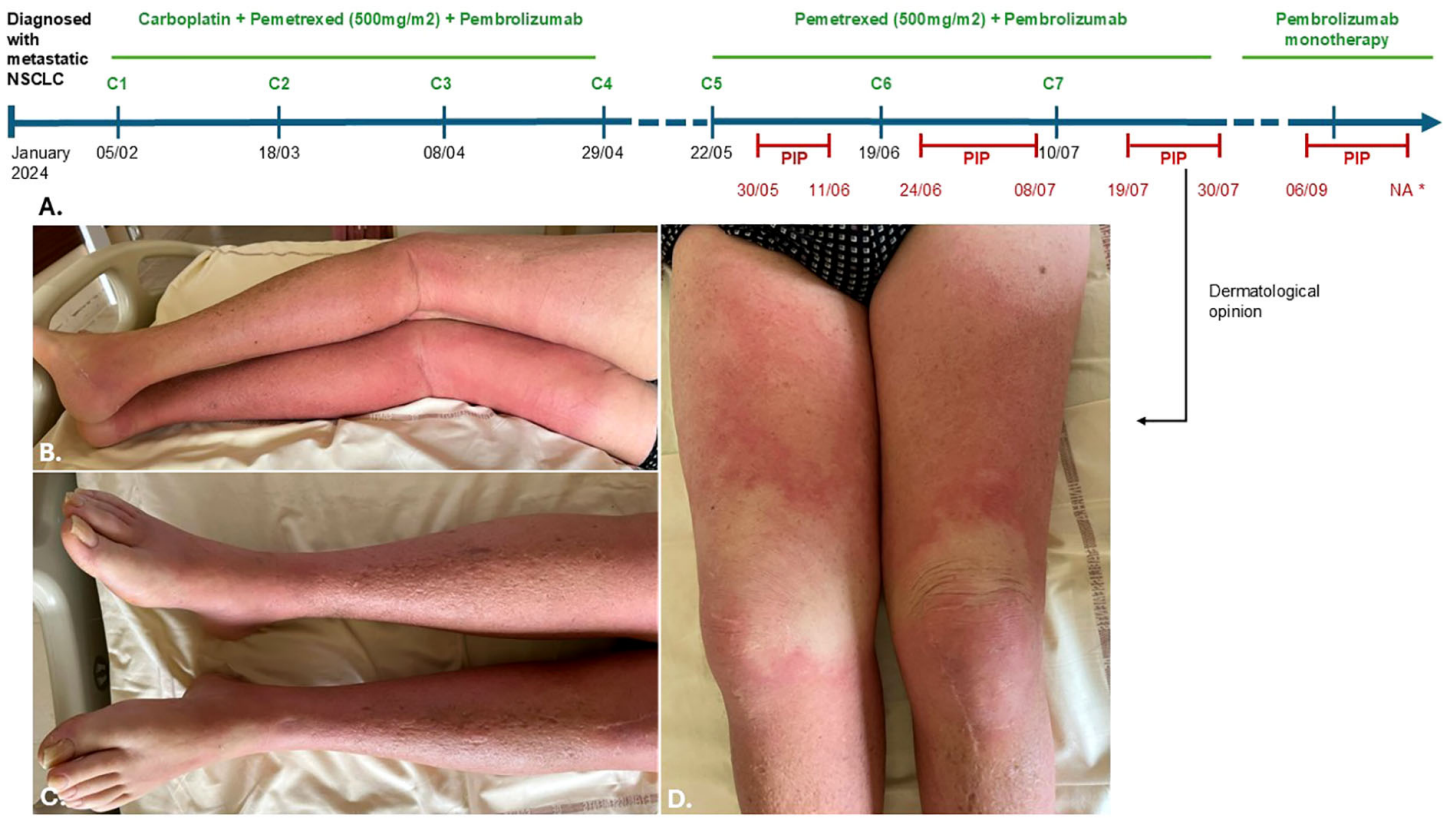
Timeline **(A)** and clinical presentation **(B–D)** of the patient’s lower limbs inflammatory edema after the seventh cycle of pemetrexed. PIP, Pemetrexed-Induced Pseudocelullitis. The red lines correspond to the inflammatory duration of each episod of PIP. Time scale generally respected, although exact distances are not strictly proportional.

An autonomous 71-year-old man, with a history of myocardial infarction, arterial hypertension, benign prostatic hyperplasia, and squamous cell carcinoma of the vocal cord (treated surgically with complete remission for 10 years), was treated for a metastatic adrenal, pleural and lymph node non-small cell lung cancer (NSCLC) with 4 cycles of carboplatin, pemetrexed and pembrolizumab combination therapy from February to April 2024. Vitamin B12, folinic acid, and prednisone were initiated alongside pemetrexed to support chemotherapy. Given a stable disease, he began pemetrexed (500mg/m2) and pembrolizumab as maintenance treatment every 3 weeks in May 2024. Eight days after the fifth dose (cumulative dose: 4962,5mg), he presented with a febrile erysipelas of the left leg, with poor response to home antibiotics, leading to hospitalization. He was treated with amoxicillin-clavulanate for 17 days. Five days after the sixth dose (5962,5mg), he was readmitted to hospital for bilateral recurrence, inflammatory edema, fever, and heart failure, and received another 15 days of amoxicillin-clavulanate. Nine days after the seventh dose (6962,5mg), he was hospitalized again for bilateral cellulitis of the lower limbs. C-reactive-protein was elevated to 250mg/l without hyperleukocytosis. Blood cultures were sterile. A CT-scan showed superficial tissue infiltration, extending into the underlying muscle layers. After 6 days of amoxicillin-clavulanate and clindamycin without improvement, a dermatological opinion was sought. Clinical examination revealed bilateral painful infiltrated oedema of the lower limbs with symmetrically distributed erythema extending to the mid-thighs, sparing the knees and soles (**Figure 1**). Based on the clinical presentation and the timeline (**Figure 1**), Pemetrexed-induced pseudocellulitis (PIP) was diagnosed. Skin biopsy showed superficial and reticular dermis with fibrosis and discrete dermal inflammatory infiltrate with lymphocytes. The clinical presentation (particularly its bilateral nature without any contributing factors) and the persistence despite several courses of appropriately administered antibiotic therapy, did not support a diagnosis of infectious cellulitis or erysipelas. The absence of blood and histological hypereosinophilia ruled out Wells syndrome. The patient had no history of venous or lymphatic lipodermatosclerosis in the hypothesis of an inflammatory flare-up, and the distribution of the lesions with intervals of healthy skin did not support this diagnosis. The Naranjo score for pemetrexed was calculated as 9, indicating a definitive adverse drug reaction. Ongoing treatments at the time of PIP included aspirin, bisoprolol, amlodipine/perindopril, rosuvastatin, and tadalafil (ongoing for over a year), as well as lansoprazole (initiated six months earlier). Although none were considered directly causally related, potential interactions with these medications may have contributed to the onset of this rare pemetrexed-related toxicity.

Pemetrexed was discontinued. An anti-inflammatory symptomatic treatment was initiated, based on evidence from previously published case reports, with oral prednisone at a dose of 20 mg per day for 5 days, combined with topical clobetasol propionate for 15 days and venous compression therapy. This approach led to complete resolution within 5 days of starting prednisone. A single recurrence occurred 3 weeks later and resolved after resumption of the same protocol. Pembrolizumab was continued alone and is currently still ongoing with stable disease.

## Literature review and discussion

A review of the literature on Pubmed and Scopus identified 47 cases of PIP in 23 articles (5–27). We included articles written in English, French and Chinese languages. Data extraction was carried out by one person (MP). All cases are summarized in a **Supplementary Table**. Four cases had no analyzable data (8), meaning 44 patients (with our patient) were included in our analyses. The data were analyzed descriptively and results are reported in **Table 1** and compared with the specific data from our case.

**Table 1:** Descriptive analysis of 44 reported cases of pemetrexed-induced pseudocellulitis in Scopus and Pubmed, including our case.

Analysed data		Results (%)	Nb of unavailable data *	Our case
Population	Sex	Male: 29 - Female: 15 - Sex ratio M/F: 1,9		M
	Age - median	64 years	14	71
	Cancer	43 NSCLC 1 endometrial ADC		NSCLC
Pemetrexed treatment	Combined systemic treatment(s)	No: 22 cases (50) Yes: 22 cases (50) - chemotherapeutic agents, antiPD1 or anti VEGF		Yes
	Nb of cycles**	Median: 5 - Extreme values: 1-40	1	5
	Time to onset after the last cycle (days)	Median: 3 - Extreme values: 2-15	33	5, 8, 9
	Dosage	500mg/m2: 15 (71,4) 400mg/m2: 1 (4,8) 375 mg/m2: 4 (19,0) 300mg/m2: 1 (4,8)	23	500mg/m2
Clinical and biological characteristics of PIP	Clinical presentation	Inflammatory edema with erythema: 43 (100) Sclerosis/induration: 23 (53,5) Pruritus: 1 (2,3)	1	Inflammatory edema with erythema
	Fever	5 (11,4)		Yes
	Location	Lower limbs: 44 (100) Associated multisite involvement (face, upper limbs, trunk): 3 (6,8)		Lower limbs
	Lesion laterality	Bilateral: 39 (88,6) - Unilateral: 5 (11,4)		uni and bilateral
	Biological inflammatory syndrome	8 (47,1)	17	Yes
	Skin biopsy performed	15 (34,1)		Yes
Management and evolution of PIP	Previous antibiotic therapy(ies)	23 (56,1)	3	Yes
	Specific treatment of PIP	None: 14 (31,8) Systemic corticotherapy: 18 (41) Topical corticotherapy: 19 (43,2)		Topical and systemic corticotherapy
	Management of pemetrexed	Discontinuation: 24 (60,0) Continuation at full dose: 7 (17,5) Continuation at reduced dose: 9 (22,5)	4	Discontinuation
	PIP evolution	Complete regression: 14 (46,7) partial regression: 14 (46,7) Persistence: 2 (6,7) Relapse: 1 (3,3)	14	Complete regression (one relapse)

Nb, number; av ,average; NSCLC ,Non-Small Cell Lung Cancer; ADC, adenocarcinoma; PD1, Programmed Death-1; NA, Not Available; PIP, Pemetrexed-Induced-Pseudocellulitis; VEGF, Vascular Endothelial Growth Factor.

*In the absence of clarification, all data were available for analysis.

**Value estimated from article data (when treatment frequency and duration were specified).

Nearly all cases involved pemetrexed treatment for NSCLC, except one case of endometrial cancer. Pemetrexed was administered either as monotherapy or in combination with other systemic agents such as chemotherapy, anti-PD1, or anti-VEGF therapies. The onset of pseudocellulitis occurred after a median of five pemetrexed doses (range 1–40), typically at a dose of 500 mg/m², as in our case. The median time from the last dose to symptom onset was variable but less than 15 days. Clinically, inflammatory edema and erythema were universally present, with frequent sclerosis, cutaneous induration, and pain; pruritus was less common. Fever and biological inflammatory syndrome were unfrequent, unlike in our case. Lesions consistently involved the lower limbs, predominantly bilaterally, while a minority of patients showed multisite involvement including the face, upper limbs, and trunk.

Skin biopsies, when performed, revealed non-specific inflammatory infiltrates, sometimes compatible with toxidermia, lipodermatosclerosis or urticarial vasculitis.

**Figure 2:**
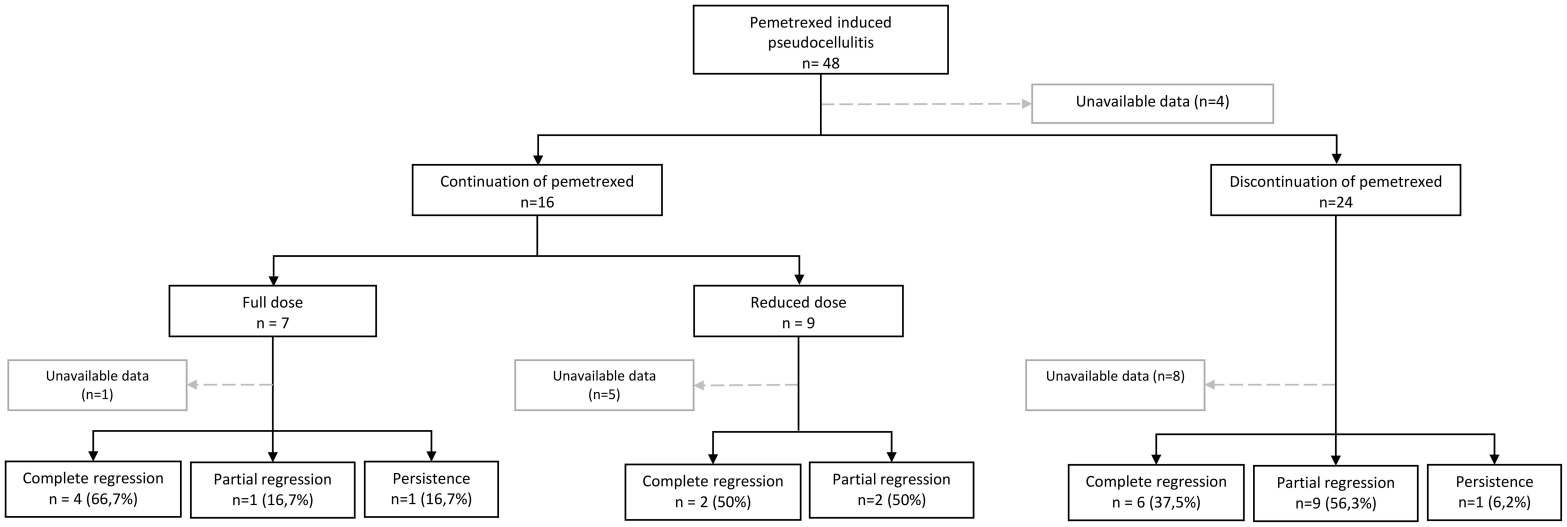
Evolution of pemetrexed-induced pseudocellulitis according to treatment continuation or discontinuation in the published literature. N, number of cases.

There is no standardized treatment for PIP (**Table 1**). More than half patients received multiple unnecessary antibiotics. Management depends on the severity of the symptoms, their impact on the patient’s quality of life, and the oncological treatment plan. Symptomatic treatment is not always essential and involve the use of topical or systemic corticosteroids. Systemic corticosteroids typically oral prednisone at variable doses and durations, were used in 41% of cases, either alone or in combination with topical corticosteroids. Among cases treated with corticosteroids, 64,3% achieved complete resolution and 35,7% partial response. Topical corticosteroids were used alone in 12 cases, showing 58,3% of partial responses, complete responses in 33.3%, and stable PIP in 8.3% (*data not shown*). **Figure 2** illustrates the evolution of PIP according to pemetrexed management. Discontinuation of pemetrexed does not always result in complete remission of PIP; cases of full remission have also been reported with continued pemetrexed at either full or reduced doses. Discontinuation of pemetrexed should not systematic and should be evaluated on a case-by-case basis through multidisciplinary discussion. Partial regression of PIP was often associated with persistent hyperpigmentation and cutaneous sclerosis. Apart from our case, no recurrence was reported after discontinuing pemetrexed.

No prophylactic measures are currently validated. Some cases reported symptom control or absence of recurrence with Pemetrexed dose reduction and/or corticosteroid treatment surrounding each chemotherapy cycle (6). Prophylactic measures, including corticosteroids and vitamin supplementation (B9, B12), have been proposed to mitigate the risk of pemetrexed cutaneous reactions (28), but need to be confirmed for PIP.

We compared our case data with those in the literature (**Table 1**). In our case report of severe pseudocellulitis, it is noteworthy that the presentation of PIP can vary within the same patient, both clinically (unilateral then bilateral) and in terms of duration since the last dose of pemetrexed. However, the clinical presentation seems to worsen over time, suggesting a cumulative-dose-effect, which we have not been able to demonstrate in the literature due to the lack of data. We decided to treat our patient with corticosteroids due to systemic reaction that had been developing for several weeks and was responsible for the decompensation of underlying conditions. While the first episodes may have improved spontaneously (with unnecessary antibiotics), the use of corticosteroids enabled a more rapid resolution of symptoms during an otherwise more severe episode. This is also the first reported case of distant recurrence despite discontinuation of pemetrexed, suggesting a possible prolonged effect over time and the need for a gradual reduction of local corticosteroid therapy.

The pathophysiology of PIP remains uncertain. It does not appear to be related to any specific pharmacological property of antifolates, as a class effect has not been observed. The absence of keratinocyte necrosis on skin biopsies, both in our case and in previously published reports, argues against a direct cytotoxic effect of pemetrexed. Similar reactions have been described with other chemotherapeutic agents such as gemcitabine (29) and taxanes (30), suggesting shared pathophysiological hypotheses including direct toxic endothelial damage, cytokine-mediated inflammation, and microvascular dysfunction (31). Pemetrexed may trigger a non-specific systemic inflammatory response leading to capillary leak syndrome. The presence of eosinophils in skin biopsies from some reported cases has led to the hypothesis of a hypersensitivity reaction. However, this cannot be confirmed in our case, as no eosinophilic infiltrate was observed histologically.

## Conclusion/perspectives

Pseudocellulitis is a rare side effect of pemetrexed that oncologists, dermatologists, and infectiologist should be aware of, to avoid unnecessary interventions, optimize patient care and prevent long-term sequelae due to prolonged inflammation. Its onset, whether from the start of pemetrexed or from the last infusion, as well as the dose, is variable. Skin biopsy is not essential for diagnosis, except to exclude a differential diagnosis. Symptomatic treatment is not always essential and is based on the use of local and/or systemic corticosteroids, with doses and duration not standardized. There appears to be a risk of distant rebound. Discontinuation of pemetrexed should not be systematic but considered on a case-by-case basis. Further research and cases are warranted to elucidate the pathogenesis, establish standardized diagnostic criteria, and develop evidence-based management strategies.

## Data Availability

The original contributions presented in the study are included in the article/**Supplementary Material**. Further inquiries can be directed to the corresponding author.

## Supplementary material

Supplementary Table 1 Summary of Pemetrexed-Induced Pseudocellulitis cases from the literature (PubMed, Scopus).
