# Supplementary material for: Case Report: Pemetrexed-induced pseudocellulitis: a rare adverse effect to be recognized for better management
Source: Front Oncol. 2025 Oct 8;15:1619250. doi: 10.3389/fonc.2025.1619250 (PMC12540078; doi:10.3389/fonc.2025.1619250)
Supplement: Supplementary Table 1 — Summary of Pemetrexed-Induced Pseudocellulitis cases from the literature (PubMed, Scopus). [file Table1.docx]

## Supplementary table . Summary of Pemetrexed-Induced Pseudocellulitis cases from the literature (PubMed, Scopus)

|  | **Patient's characteristics** | | | **Pemetrexed treatment** | | | **Clinic and investigations** | | | | | **Management and evolution of PIP** | | | | |
| --- | --- | --- | --- | --- | --- | --- | --- | --- | --- | --- | --- | --- | --- | --- | --- | --- |
| **First author,  year** | **No** | **Age/ Sex** | **Cancer type** | **Associated  treatment** | **No of doses  (duration)** | **Time to onset  post-dose (days)** | **Fever** | **Localisation** | **Laterality** | **Inflammatory syndrome** | **Biopsy** | **Antibiotics** | **Specific treatment** | **Pemetrexed  status** | **Clinical outcome** | **Time to  improvement (days)** |
| **Our Case** | 1 | 70/M | NSCLC | Carboplatin + anti-PD1,  then anti-PD1 | 5 | 8 | Yes | lower limbs | Bilateral | Yes | Yes | Yes | Prednisone 20mg/d for 5 days and local corticostreroïds | Stopped | CR | 30 |
| **Nguyen, 2025** | 1 | 58/F | NSCLC | - | NA (3 mos) | NA | No | lower limbs | Bilateral | NA | Yes | Yes | Prednisone 10 mg/d  and local corticostreroïds | NA | PR | NA |
| **Horton, 2024** | 2 | 60/M | NSCLC | - | 9 | 3 | No | lower limbs | Bilateral | No | No | Yes | Prednisone 40mg/d for 8 days  and after each dose | Continued (FD) | CR | NA |
|  |  | 47/M | NSCLC | Loratinib | 11 | 3 | No | lower limbs | Bilateral | NA | Yes | Yes | Prednisone 20 mg/d + local corticostreroïds 2/d | Continued (FD) | CR | NA |
| **Yan, 2023** | 1 | 44/M | NSCLC (ADC) | Cisplatin | 1 | 3 | Yes | lower limbs | Unilateral | Yes | No | Yes | Prednisone 30mg/d  + antihistaminic | Continued (RD) | PR | 90 |
| **Doyle, 2023** | 1 | 54/M | NSCLC (ADC) | Radiation, carboplatin, anti-PD1,  then radiation, anti-PD1, bevacizumab | NA (37 mos) | NA | No | lower and upper limbs face lower back | Bilateral | No | No | Yes | Local corticostreroïds 2/d | Stopped | PR | 60 |
| **Pach, 2023** | 4 | NA | NA | NA | NA (NA) | NA | NA | NA |  | NA | NA | NA | NA | NA | NA | NA |
| **Sánchez, 2020** | 1 | 59/F | NSCLC (ADC) | - | 40 (36 mos) | 2 | No | lower limbs | Bilateral | Yes | Yes |  | Local corticostreroïds | Sopped | PR | 60 |
| **Sarrazin, 2020** | 1 | 63/M | NSCLC (ADC) | Cisplatin, Bevacizumab | 20 | NA | No | lower limbs | Bilateral | No | Yes | Yes | Local corticostreroïds | Stopped | CR | 90 |
| **Lo, 2020** | 1 | 75/M | NSCLC | Cisplatin | 7 | 4 | No | lower limbs | Bilateral | Yes | No | Yes | Prednisonolone 20mg/day for 15 days  + local corticostreroïds 2/d for 3 days | Sopped | CR | NA |
| **Liau, 2017** | 1 | 66/M | NSCLC | Cisplatin | 2 | 8 | No | lower limbs | Unilateral | No | Yes | Yes | Local corticostreroïds 2/d | Continued (FD) | CR | 21 |
| **Wollina, 2017** | 1 | 62/M | NSCLC | Cisplatin | NA (6-8 mos) | 15 | No | lower limbs | Bilateral | Yes | Yes | Yes | Prednisolone 80mg/d, local corticostreroïds, B vitamins | Stopped | PR | 10 |
| **Santosa, 2017** | 1 | 79/M | NSCLC | Carboplatin | 5 | NA | No | lower limbs, Abdomen, Flanks | Bilateral | No | Yes | Yes | Local corticoïds and gentamicin 0.1% | Stopped | CR | 60 |
| **Tracey, 2016** | 1 | 61/F | endometrioid  endometrial  ADC | - | 1 | NA | No | lower limbs | Bilateral | NA | Yes | NA | Oral prednisone and local corticostreroïds | Stopped | PR | NA |
| **Ishikawa, 2016** | 1 | 63/M | NSCLC (ADC) | - | 18 | NA | No | lower and upper limbs Face Chest | Bilateral | NA | Yes | No | None | Continued (FD) | Stable | - |
| **Corbaux, 2015** | 2 | 66/M | NSCLC (ADC) | Cisplatin | 6 | NA | No | lower limbs | Bilateral | NA | No | No | Local corticostreroïds | Sopped | PR | NA |
|  |  | 70/F | NSCLC | Carboplatin,  Gemcitabin | 1 | NA | No | lower limbs | Bilateral | NA | No | Yes | Local corticostreroïds | Sopped | PR | NA |
| **Shuster, 2015** | 3 | 75/M | NSCLC | - | 16 * (11 mos) | NA | No | lower limbs | Bilateral | NA | No | No | Local corticostreroïds 2/d | Stopped | PR | NA |
|  |  | 57/F | NSCLC | - | 20 * (14 mos) | NA | No | lower limbs | Unilateral | NA | Yes | Yes | Local corticostreroïds 2/d | NA | PR | NA |
|  |  | 66/F | NSCLC | - | 3 * (2,5 mos) | NA | No | lower limbs | Unilateral | NA | No | NA | Local corticostreroïds 2/d | Continued (FD) | CR | NA |
| **Merklen-Djafri, 2012** | 3 | 57/M | NSCLC (BAC) | - | 16 | NA | No | lower limbs | Bilateral | NA | Yes | No | None | NA | CR | NA |
|  |  | 65/M | NSCLC | - | 2 | 15 | Yes | lower limbs | Bilateral | NA | No | No | None | Continued (RD) | PR | NA |
|  |  | 62/M | NSCLC (BAC) | - | 7 | NA | No | lower limbs | Bilateral | NA | No | No | None | Stopped | PR | NA |
| **Salle De Chou, 2012** | 1 | 83/M | NSCLC (BAC) | - | 1 | 2 | Yes | lower limbs | Bilateral | NA | Yes | Yes | Local corticostreroïds | Stopped | Stable | - |
| **Katsenos, 2012** | 1 | 70/F | NSCLC (ADC) | - | 4* (3 mos) | NA | No | lower limbs | Bilateral | NA | No | NA | Systemic corticostreroïds | Continued (RD) | CR | NA |
| **D'Angelo, 2012** | 14 | 72 (av.)/ M (7) F(7) | NSCLC (ADC) | Combination (9) : bevacizumab, cisplatin/carboplatin, paclitaxel, or cetuximab Monotherapy (5) | 8 median, range, 3–23 | NA | No | lower limbs | Bilateral | No | No | Yes (6) | Prednisone 20 mg/d for 5 days (4) None (10) | Stopped (8) Continued (6) (1 FD and 5 RD) | NA | NA |
| **Vitiello, 2011** | 1 | 36/F | NSCLC | - | 1 | 3 | No | lower limbs | Bilateral | Yes | Yes | Yes | Local corticostreroïds and antihistaminic 3/d | Continued (FD) | PR | 2 |
| **Eguia, 2011** | 1 | 67/M | NSCLC (BAC) | - | 15 | 3-10 | Yes | lower and upper limbs Face | Bilateral | Yes | No | No | Prednisone 0.5 mg/kg/d for 14 days | Stopped | PR | NA |
| **Galetta, 2011** | 3 | 59/M | NSCLC | Cisplatin | 4 | NA | No | lower limbs | Bilateral | NA | No | Yes | Oral and topical corticostreroïds | Stopped | CR | 30 |
|  |  | 65/M | NSCLC | Cisplatin | 2 | NA | No | lower limbs | Unilateral | NA | No | No | Systemic corticostreroïds | Stopped | CR | 20 |
|  |  | 66/M | NSCLC | - | 3 | NA | No | lower limbs | Bilateral | NA | No | No | Systemic corticostreroïds | Continued (RD) | CR | NA |
| **Lopes, 2006** | 1 | 68/M | NSCLC | - | 2 | 15 | No | lower limbs | Bilateral | Yes | Yes | Yes | Prednisone 1mg/kg for 3 days | NA | CR | 3 |
|  |  |  |  |  |  |  |  |  |  |  |  |  |  |  |  |  |

*No : number; PIP Pemetrexed-Induced-Pseudocellulitis; av : average ; NSCLC : Non-Small Cell Lung Cancer; ADC : adenocarcinoma, BAC : Bronchioloalveolar Carcinoma; PD1: Programmed Death-1; FD: Full-dose; RD: Reduced-dose; /d : per day, Mos : months; CR : complete resolution; PR : partial resolution*

*NA : Not Available:*

** Value estimated from article data (when treatment frequency and duration were specified)*
